# RNA Hydrogel Combined with MnO_2_ Nanoparticles as a Nano-Vaccine to Treat Triple Negative Breast Cancer

**DOI:** 10.3389/fchem.2021.797094

**Published:** 2021-12-24

**Authors:** Weicai Wang, Xiaofan Liu, Lairong Ding, Hyung Jong Jin, Xuemei Li

**Affiliations:** ^1^ Collaborative Innovation Center of Tumor Marker Detection Technology, Equipment and Diagnosis-Therapy Integration in Universities of Shandong, Shandong Province Key Laboratory of Detection Technology for Tumor Makers, School of Chemistry and Chemical Engineering, Linyi University, Linyi, China; ^2^ Department of Bioscience and Biotechnology, The University of Suwon, Hwaseong, South Korea

**Keywords:** RNA hydrogel, nano-vaccine, triple negative breast cancer, gene therapy, photodynamic therapy

## Abstract

Hypoxia is not only the reason of tumor metastasis but also enhances the spread of cancer cells from the original tumor site, which results in cancer recurrence. Herein, we developed a self-assembled RNA hydrogel that efficiently delivered synergistic DNA CpG and short hairpin RNA (shRNA) adjuvants, as well as MnO_2_ loaded-photodynamic agent chlorine e6 (MnO_2_@Ce6), and a chemotherapy drug doxorubicin (DOX) into MDA-MB-231cells. The RNA hydrogel consists of one tumour suppressor miRNA (miRNA-205) and one anti-metastatic miRNA (miRNA-182), both of which showed an outstanding effect in synergistically abrogating tumours. The hydrogel would be dissociated by endogenous Dicer enzyme to release loaded therapeutic molecules, and in the meantime induce decomposition of tumor endogenous H_2_O_2_ to relieve tumor hypoxia. As a result, a remarkable synergistic therapeutic effect is achieved through the combined chemo-photodynamic therapy, which simultaneously triggers a series of anti-tumor immune responses. Besides, the hydrogel as the carrier which modified aptamer to targeted MDA-MB-231 has the advantages of good biocompatibility and low cytotoxicity. This strategy could be implemented to design any other microRNA (miRNA) as the carrier, combined with other treatment methods to treat human cancer, thereby overcoming the limitations of current cancer therapies.

## Introduction

Triple negative breast cancer (TNBC) has a high rate of metastasis and a poor prognosis, accounting for 10–20% of all breast cancers (Ismail-Khan and Bui, 2010; Castro et al., 2015; Liu et al., 2018; Gong et al., 2021). The treatment of patients with TNBC remains a great clinical challenge due to the poor prognosis resulting from the recurrence and metastasis (Lee et al., 2019; Li et al., 2019; Ji et al., 2021). The disadvantages of the conventional treatment of TNBC are poor cell uptake, poor bioavailability, and resistance (Pawar and Prabhu, 2019). With the outstanding contribution of nucleic acid nanotechnology in biological medicine (Seeman, 2010; Shu et al., 2011; Cutler et al., 2012; Zhu et al., 2013a; Zhu et al., 2013b; Jang et al., 2015; Yue et al., 2019; Yu K.-X. et al., 2021), the synthesis of functional nucleic acid as the targeting delivery treatment agent has become a popular transfer type for the integration of diagnosis and treatment of TNBC (Peng et al., 2020; Yu K. et al., 2021; Yue et al., 2021; Zhang et al., 2021). However, there are many challenges for the therapeutic application of miRNA, such as targeting, systemic delivery, and susceptibility to biodegradation. RNA hydrogel is biocompatible, has designable mechanical properties, and exhibits a sensitive response to external stimuli such as temperature, light, enzyme, DNA, pH, and small molecules (Zhu et al., 2017). In our previous work, RNA hydrogel, as nano-vaccine, has been actively explored for delivering immunomodulatory adjuvants and antigens for cancer immunotherapy, and shows great potential in the treatment of TNBC (Ding et al., 2020; Li et al., 2020). However, the delivery of only nucleic acid drugs significantly limits the further application of RNA hydrogels in human cancer detection and treatment. Therefore, it is very urgent to implement new treatment strategies using RNA hydrogel delivery system, combined with other treatment methods to overcome current limitations of cancer therapies.

Hypoxia is not only the reason of tumor metastasis but also enhances the spread of cancer cells from the original tumor site, which causes the growth of next tumors and results in cancer recurrence (Wilson and Hay, 2011). Besides, due to the rapid proliferation of cancer cells and the distortion of cancer blood vessels, the hypoxic nature of the cancer microenvironment was highly detrimental to the oxygen-dependent PDT (Chen et al., 2015). Many methods have been reported to increase the oxygen concentration in the tumor microenvironment. For example, artificial blood substitutes such as perfluorocarbon-based oxygen carriers have been used to transport oxygen into the tumor (Krafft, 2020; Mai et al., 2021). This method may be limited by the distance from the tumor. Recently, the use of manganese dioxide to modify the hypoxic microenvironment of tumors has received widespread attention (Tao et al., 2018; Yin et al., 2021). Through the reaction of MnO_2_ dioxide with endogenous hydrogen peroxide in the tumor microenvironment, MnO_2_ was degraded to generate O_2_ and Mn^2+^. The released O_2_ can enhance the subsequent photodynamic therapy which can precisely ablate local cancers through the strong oxidative capacity of reactive oxygen species (ROS) on nucleic acids, enzymes, and cellular membranes (Secret et al., 2014). In addition, a large number of literatures have reported that the use of photosensitizer to improve the tumor microenvironment and photodynamic therapy can greatly enhance the damage of agents on tumor tissue, alter the weak acidic environment, and further consolidate the metastasis and recurrence of cancer tumor (Yang et al., 2015; Chen et al., 2016; Zhu et al., 2016).

Inspired by these findings, we designed a novel nano-vaccine that used the electrostatic combination of RNA hydrogel and manganese dioxide nanoparticles to treat triple-negative breast cancer. According to the research by Joao Conde (Conde et al., 2016), Avital Gilam (Gilam et al., 2016), and others, we used miRNA-205 and miRNA-182 to inhibit the growth and metastasis of MDA-MB-231 cells. The hydrogel after cholesterol (chol) compression has a multi-copy structure and contains multiple G-C sites, which will provide a large number of sites for DOX as a chemical treatment drug. The obtained hydrogel/DOX (HD) can be cleaved by Dicer enzyme in cells, and the multi-copy structure can release CpG fragments as immune genes as well as miRNA-205, miRNA-182, and DOX to kill cells. The developed nano-platform allowed for not only targeted gene, chemotherapy, and on-demanding drug release, but also improving the tumor microenvironment and photodynamic therapy. As a result, a remarkable *in vitro* or *in vivo* therapeutic effect was achieved through the combination of multiple therapies, and the hydrogel triggered immune responses, and was cut off by Dicer enzyme and then released gene, DOX and MnO_2_@Ce6 (MC). Under the release of oxygen, the photosensitizer generated singlet oxygen under 660 nm laser stimulation, thereby enhancing photodynamic therapy. In addition, the irradiation of laser also increased the medicinal properties of DOX, so that DOX quickly accumulated in the cell nucleus, thereby realizing the photodynamic therapy and chemotherapy and double gain nano diagnosis platform. This work was creatively combined with gene therapy, chemical treatment, and photodynamic therapy, improved the tumor microenvironment through the release of oxygen, and gained three types of combination therapies.

## Experimental Section

### Materials

KMnO_4_ was purchased from Triad chemical reagent Co., Ltd. Ce6 was obtained from Frontier Scientific (Logan, UT, United States). PAH (Mw∼17500) was purchased from Macklin. EDC and NHS were from Aladdin. T7 RNA polymerase was purchased from New England Biolabs. T4 DNA ligase was obtained from Thermofisher Scientific. In all the experiments, water (18.25 MΩ) was sterilized at high temperature. DOX was from Sangon Biotech Co., Ltd.

MDA-MB-231 (human breast cancer cell lines) was purchased from Procell Life Science&Technology Co., Ltd. MCF-7 (human breast cancer cell lines) was purchased from American Type Culture Collection (ATCC, Manassas, VA). DNA/RNA sequences (Supplementary Table S1) were obtained from Takara Biotechnology (Dalian) Co., Ltd. and Genscript Biotechnology (Nanjing) Co., Ltd.

### Programmable Self-Assembly of RNA Hydrogel

We designed two single-stranded DNA, i.e., a therapeutic ssDNA including the anti-sequence of miRNA-205 and miRNA-182 and a scrambled miRNA designed in the control group. T7 promoter and ssDNA were added to TM buffer (30 mM Tris-HCl with the pH of 7.8 mixing with 10 mM MgCl_2_) to obtain the final concentration of 0.25 μM. Then, the mixture was heated to 95°C for 5 min and then slowly cooled down to 25°C. After adding T4 ligase (1U/10 μl) and T4 ligase buffer, the mixed solution was placed at 19°C for 13 h. The cut circular DNA/T7 promoter chimeras would change to a closed-loop circular DNA/T7 promoter. Then, T7 polymerase (1 µ/10 μl), T7 polymerase buffer, and rNTP were added and mixed, and the reaction lasted for 5 h at 37°C in a constant-temperature chamber. After the polyreaction reaction, the F-C-LXL apt and CpG strands were added in the reaction system, obtaining the final concentration of 0.45 μM. The reaction system was heated up to 65°C, maintained at the temperature for 5 min, and slowly cooled to 25°C. Then, the system was quickly transferred to a 4°C refrigerator and kept there for 2 h. The resultant products were washed with DI water at the ratio of 1:1. The mixture was centrifugated at 8000 rpm for 5 min, and the supernatant was extracted. The centrifugation and extraction processes were performed twice. The obtained hydrogel was mixed with DOX with the concentrations of 20, 40, 60, 80, 100, and 150 μM for 2 h at 37°C. Then, the mixture was washed with DI water and the fluorescence intensity of the extracted supernatant was measured. Meanwhile, the fluorescence intensity of DOX at the same concentration was measured. The differences in the fluorescence intensity between hydrogel-DOX mixture and DOX at 626 nm were obtained. When the differences reached stability, the hydrogel achieved saturation. The obtained samples were stored at 4°C for the subsequent characterization.

### Synthesis of MnO_2_@Ce6

MnO_2_ was synthesized according to the published methods (Prasad et al., 2014; Song et al., 2016). A total of 64 mg of KMnO_4_ was dispersed in 20 ml of DI water, and 37.5 mg of PAH was dissolved into 1 ml of DI water. We placed 900 μl KMnO_4_ and 100 μl PAH into 1.5 ml centrifuge tube for at least 15 min at room temperature till all permanganate was converted into colloidal MnO_2_, which was confirmed by UV-vis (Nanodrop 2000). Then, the mixture was put into 50 μl NaCl (1 M) and centrifuged at 16500 (KDC-140HR) by high speed refrigerated centrifuge for 1 h. The 500 μl supernatant was collected and stored at 4°C for the next characterization.

Ce6 was introduced onto colloidal MnO_2_ through covalent bond, and before that, Ce6 was activated by EDC and NHS in DMSO. Ce6 (1 ml, 20 mg/ml) in DMSO was mixed with 6.4 mg of EDS and 4.6 mg of NHS in dark at room temperature for 20 min. The supernatant of 20, 40, 60, and 80 μl colloidal MnO_2_ was added into 20 μl of activated Ce6 (10 mg/ml) with ultrasonication for 4 h. The excessive unbound Ce6 was removed by high speed refrigerated centrifuge for 10 min.

### Synthesis of Hydrogel-MnO_2_@Ce6 and Hydrogel/DOX-MnO_2_@Ce6

MnO_2_@Ce6 was unstable in FBS, while hydrogel modified with cholesterol was stable in FBS (Jang et al., 2016). Hydrogel had negative zeta potential, while MnO_2_@Ce6 had positive charge; thus, the MnO_2_@Ce6 can be introduced onto hydrogel by electrostatic interaction. The obtained MnO_2_@Ce6 and hydrogel were diluted and mixed together, and the resultant product was Hydrogel-MnO_2_@Ce6 (HMC). When they reached saturation, MnO_2_@Ce6 would not condense. The same method was used to synthesize Hydrogel/DOX-MnO_2_@Ce6 (HDMC).

### Cellular Experiments

All cells were cultured in DMEM medium (HyClone, HIGH GLUCOSE) containing 10% FBS (Gibco) and 1% penicillin-streptomycin (Gibco) at 37°C in a CO_2_ incubator (Thermo, 3111) with 5% CO_2_ and 95% air humid atmosphere. After the cell density reached 85%, they were separated into two or three plates by Trypsin-EDTA (HyClone, 0.25%). Prior to each experiment, the cell density was determined by a hemocytometer.

In order to examine the cellular uptake of hydrogel and other nanoparticles, MDA-MB-231 cells were planted in glass bottom cell culture dish (1×10^5^/dish). After the cells adhered to the bottom, 10 μl hydrogel or other nanoparticles were added to incubate MDA-MB-231 cells. Two hours later, the cells were stained with DAPI and washed 3 times by PBS buffer. In addition, 1 ml PBS was added before laser confocal imaging.

For flow cytometry assay, MDA-MB-231 cells were seeded in 6-well plates, and remixed with hydrogel, HD, MnO_2_, MnO_2_@Ce6, HMC and HDMC for 2 h. Then MnO_2_@Ce6, HMC and HDMC were exposed to 660 nm irradiation at a power density of 50 mW cm^−2^ for 10 min. Then the cells were collected in 1.5 ml tube and its fluorescence signals were tested.

For cell toxicity assay, MDA-MB-231 cells were seeded in 96-well plates for at least 12 h. After consuming hydrogel, HD, MnO_2_, MnO_2_@Ce6, and HMC, the survival of MDA-MB-231 cells was measured by CCK-8, which was purchased from DOJINDO. The pure cultures were used as the blank group, the pure cells were used as the control group, and the experimental group was added with 10 μl hydrogel, HD, MnO_2_, MnO_2_@Ce6, HMC, and HDMC. The total volume of each well in the 96-well plate was 100 μl. After MDA-MB-231 cells (1×10^4^ cells/well) adhered to the plate completely, the 96-well plate was placed into an incubator of 5% CO_2_, 95% air humid for 24 h at 37°C, and then the hydrogel and other nanoparticles were added and incubated for 12, 24, 36, 48, and 56 h. After 55 min, the formazan was formed, and its absorbance was tested by enzyme-linked immunometric meter (DG5033A). Enzyme-linked immunometric meter measured the absorbance at 450 nm. Three tests were performed for each group (cell survival rate = (OD_test_ - OD_blank_)/(OD_control_ - OD_blank_)).

For photodynamic therapy, MDA-MB-231 cells were seeded in 96-well plates and mixed with HMC or HDMC at various time stages. After 2 h, the 96-well plates were exposed to 660 nm irradiation at a power density of 50 mW cm^−2^ for 10 min. Then, the cells were transferred into fresh media and further incubated for 6, 12, 24, 36, and 48 h. After different incubation time, 10 μl CCK-8 was added in each well, and the culture plate was tapped to mix well. After 55 min, the formazan was formed, and its absorbance was tested by enzyme-linked immunometric meter (DG5033A). Enzyme-linked immunometric meter measured the absorbance at 450 nm. Three tests were performed for each group.

### Animal Model

In order to determine the biological and pathological relevance of the inhibitory effects of hydrogel, HD, MnO_2_, MnO_2_@Ce6, HMC, and HDMC (with or without light) to the anticancer effects on 4T1 cells [4T1 cells were selected because tumour growth and metastatic spread of these cells in BALB/c female mice can closely mimic Stage IV human breast cancer (Luo, 2007)], we analyzed in female Balb/c mice weighting 16–18 g. The mice were purchased from Chongqing Medical University (Chongqing, China) and used in accordance with animal regulations provided by Linyi University Laboratory Animal Centre. 4T1 solid tumours were subcutaneously inoculated into the back of these mice by injecting 1×10^6^ cells/100 μl of serum-free PBS. All the mice were fed with normal diet (research diet) containing carbohydrate (64.0%), protein (20.0%), and fat (16.0%) with total energy of 3941 kcal/kg. The room for the mice had abundant water and standard food which had been treated by high-pressure sterilizer (LDH-100KBS) at high temperature, and the room kept the cycle of 12 h of light and 12 h of dark environment. When the volume of tumours reached around 100 mm^3^, the mice bearing 4T1 tumours were treated. The tumour sizes and mice body weights were recorded in the following 14 days. After 14 days, the tumours of all nude mice were collected and photographed. Moreover, we collected hearts, livers, kidneys, spleens, lungs, and intestines from the mice, and stored them in triformol (4%) at room temperature for the immunohistochemical analysis. Both tumours and viscera were analyzed by H and E straining to detect the degree of disease, and all tumours were tested for their cell proliferation by TUNEL. The size of individual tumour was measured by a Vernier caliper (AIRAJ), and the volume of tumour was calculated by the formula Vtumour (mm^3^) = width×length^2^× 0.5.

## Results and Discussion

### Synthesis and Characterization of HDMC

**FIGURE 1:**
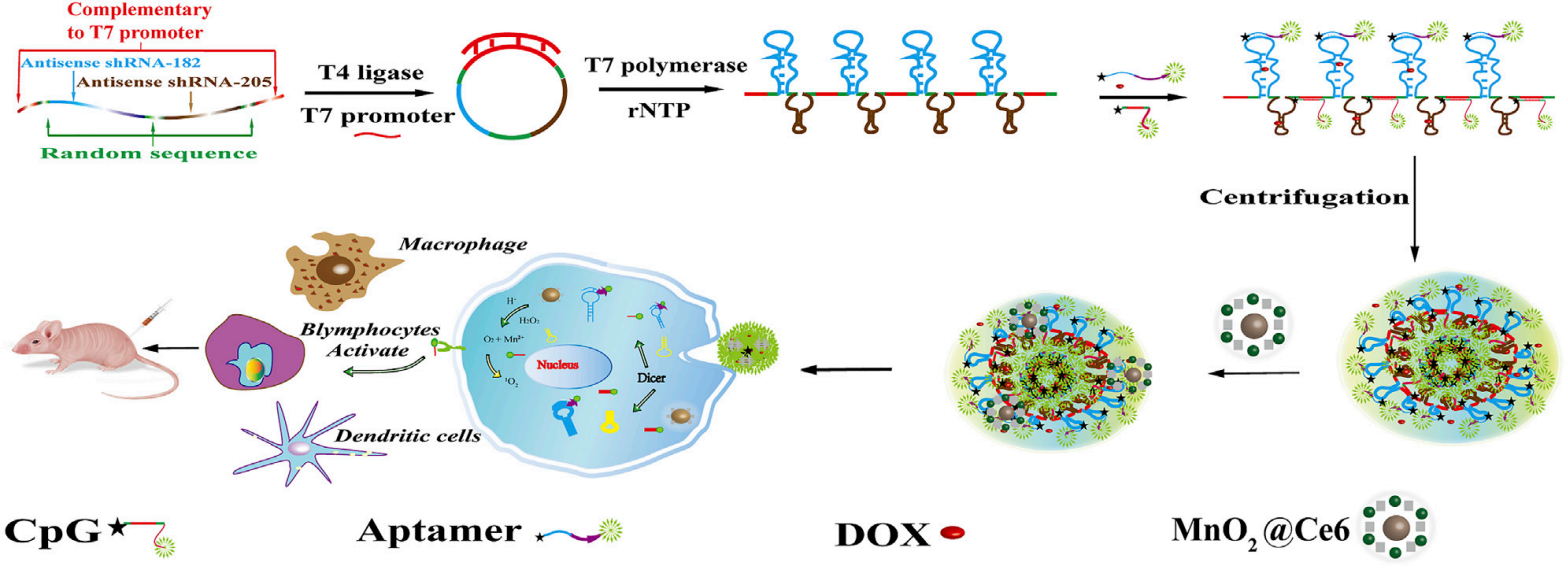
Schematic illustration of self-assembly of HDMC for targeted drug delivery in cancer cells. After vitro assays, intro assay was set up to analyze the immunoreaction of the HDMC and the microenvironment.

The synthesis procedure of hydrogel and HD, hydrogel/DOX-MnO_2_@Ce6 is illustrated in Figure 1. To synthesize the two-in-one RNA nano-hydrogel for simultaneous co-delivery of miRNA-182 and miRNA-205, we first designed single-stranded DNA template, which would be transcribed to RNA copy transcripts by rolling circle transcription (RCT). The linear single-stranded DNA template (ssDNA) was composed of antisense miRNA-182 sequence and antisense miRNA-205 sequence, random sequence for interrupting antisense miRNA-182 sequence and antisense miRNA-205 sequence, and T7 promoter primer-binding sites. The linear ssDNA template that both ends were complementary to T7 promoter was annealed with T7 promoter primer, resulting in circular DNA with a nick. After DNA ligase connected the nicked sequence, T7 RNA polymerase generated the polymerized transcripts (that is, Rtrs) from the closed circular DNA template through RCT, which contained multiple tandem copies of miRNA-182 sequence and miRNA-205 sequence. Next, we designed CpG-chol and aptamer (apt)-chol bond to these transcripts. The cholesterols would be tightly packed under the limited space because of hydrophobic interactions (Xiao et al., 2011); then, the supernatant was removed after high-speed centrifugation, and the RNA hydrogel was finally obtained. After cleavage by dicer enzyme, miRNA and CpG would be uptake by cells. Moreover, because there are a large number of packed DNA base pairs, the RNA Nano hydrogels spatially facilitate for cargo loading, especially for chemotherapeutic drug DOX that can be preferentially introduced into double-stranded GC or CG base pairs.

**FIGURE 2:**
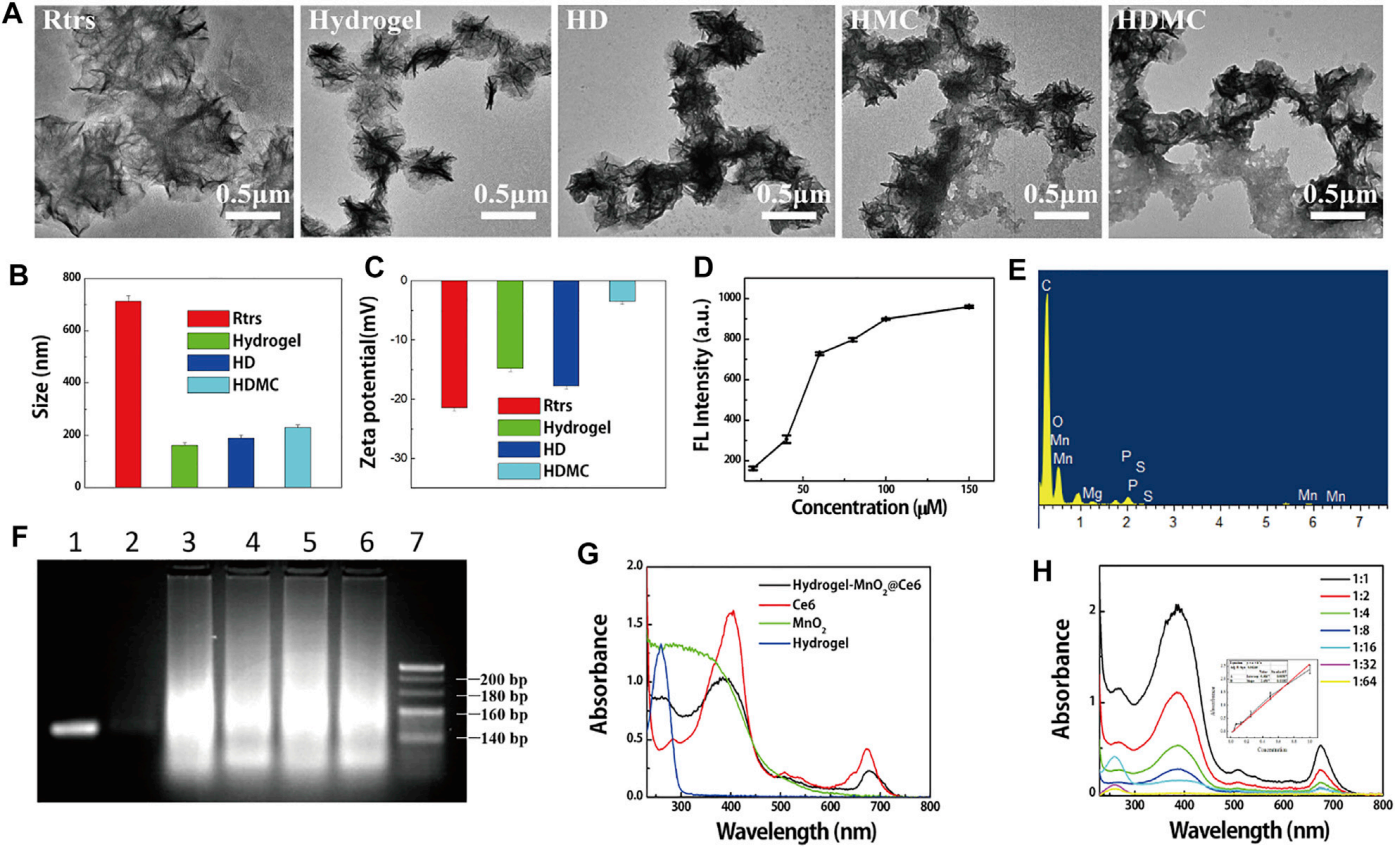
Characterization of HDMC. **(A)** Transmission electron microscopy images showing size and morphology of particles. **(B)** Hydrodynamic size distribution and **(C)** zeta potential of Rtrs, hydrogel, HD, HDMC. **(D)** The different values of fluorescence intensity between different concentration of DOX (20, 40, 60, 80, 100, and 150 μM) and the supernatant of DOX-loading on hydrogel. **(E)** EDS of HDMC. **(F)** AGE (agarose gel electrophoresis) assay of ssDNA (1), Rtrs (3), Rtrs-apt (4), Rtrs-CpG (5), hydrogel (6), and circular DNA with a nick (circle) (2), 500 bp marker (7). Nucleic acids were visible under ultraviolet irradiation after EB staining. **(G)** The UV-vis spectrum of HMC, Ce6, MnO_2_, and hydrogel was illustrated and **(H)** was the different concentration of MC, and we could calculate the loading rate of MC onto HMC from the linear equation *y* = 4.259x−0.021 inserted.

The synthesis procedure of MnO_2_@Ce6 is illustrated in Supplementary Figure S1A. Colloidal MnO_2_ was first produced according to the literature methods. In brief, 64 mg of potassium permanganate (KMnO_4_) was reduced to colloidal MnO_2_ because of the cationic polyelectrolyte Poly (allylamine hydrochloride) (PAH) resulting in a dark brown solution. To formulate MnO_2_@Ce6, Ce6 was activated by 1-ethyl-3-(3-dimethylaminopropyl) carbodiimide (EDC) and N-hydroxysuccinimide (NHS) in dimethyl sulfoxide (DMSO) in advance, and then mix the aqueous solution of colloidal MnO_2_ nanoparticles at different weight ratios (Ce6:MnO_2_ = 1:1, 1:2, 1:3, and 1:4) under ultra-sonication at room temperature for 4 h. The change of KMnO_4_, MnO_2_, and MnO_2_@Ce6 was illustrated by UV-vis spectra (Supplementary Figure S1B), the characteristic KMnO_4_ peaks (315, 525, and 545 nm) disappeared after this reaction, while a new broad absorbance peak around 300 nm appeared, which should be resulted from the surface plasmon band of colloidal MnO_2_ (DuPre et al., 2007). As revealed by transmission electron microscope (TEM, Supplementary Figures S1G,H), as-made colloidal MnO_2_ and MnO_2_@Ce6 showed average sizes at around 30 and 80 nm. What is more, they appeared different scattering peak and zeta potential as revealed in Supplementary Figures S1C,D, the scattering peak of MnO_2_ is around 781 nm, and its zeta potential is 33.4 mV, while MnO_2_@Ce6 is around 673 nm and its zeta potential is 0.5 mV. The dark-field microscopy of MnO_2_@Ce6 and MnO_2_ was shown in Supplementary Figures S1E,F. All of them were scattering orange. MnO_2_@Ce6 was unstable in Dulbecco’s modified eagle medium with fetal bovine serum (DMEM-FBS), which would coagulation when mix them up. The hydrogel which modified cholesterol would be stable in DMEM-FBS. Besides, hydrogel was produced by RCT which was anionic aggregates that would bond with MnO_2_@Ce6 by electrostatic interaction. The differences between Rtrs, Rtrs-apt-CpG-chol (hydrogel), and HD were detected by size and zeta potential, as illustrated in Figures 2B,C. The size of Rtrs is around 712 nm, and zeta potential is around −21.4 mV; the size of hydrogel is fall down around to 162 nm and zeta potential is around −14.8 mV; the size of HD change to 190 nm and zeta potential is around −17.8 mV. The smeared bands observed on the agarose gel demonstrated the polymerized RNA transcripts had a wide range molecular weights and excess of initial DNA strands (Figure 2F). After we mixed the DOX and hydrogel for 2 h at 37°C in shaker, washed it by double distilled water (DI water) to remove the excess DOX *via* high speed centrifuge finally got the HD.

Take 5 μl into 1 ml tube and measure its zeta potential. What is more, when DOX conjunct to the hydrogel, the zeta potential changed, as shown in Figure 2C. We measured the morphology (micro sponge) of Rtrs and HD, as shown in Figure 2A. When the Rtrs was conjugated cholesterol and DOX, the size of Rtrs reached micron level, and the hydrogel was in a compact state with the size of around 190 nm. In order to determine whether the hydrogel has already saturated with DOX, we set up two groups. In the first group, 5 μl DI water was sprinkled into different concentrations of DOX, and in the second group, 5 μl hydrogel was sprinkled into different concentrations of DOX. Then, the supernatants were collected and their fluorescence intensity was tested. The fluorescence differences between different substances with the same concentration in the two groups were calculated and the tropism was obtained, as shown in Figure 2D. From the result, the saturation concentration was determined to be 100 μM. In order to detect the loading quantity of DOX, the fluorescence spectra of different samples were recorded with a fluorescence spectrophotometer (F-460, Hitachi, Japan).

TEM was used to characterize the morphology and size of hydrogel, hydrogel-MnO_2_@Ce6, HDMC. UV–vis spectra were used qualitatively to detect the products in each stage. The zeta potentials of these products were obtained on a Malvern Zettaliter. The morphology of hydrogel is shown in Figure 2A. From the RCT, we obtained many types of similar micro-sponges, which was a strong polyanion. When MnO_2_@Ce6 had a little positive charge, we adjusted the proportion of hydrogel or HD and MnO_2_@Ce6 to obtain the HMC and HDMC which were stable in aqueous solution. From Figure 2C, we can see that the zeta potential of HDMC was higher than HD, but it was still negative. Positively charged particles are often easily sequestered by macrophages in the lung, liver and spleen, whereas negatively charged HDMC are not. From Figure 2A, there are many adhesion substances around the hydrogel and HD. From Supplementary Figure S2A and Figure 2E, the elemental analysis of HMC and HDMC shows that there are manganese and oxygen elements, which confirms that the hydrogel can carry MC. The carbon and phosphorus elements come from hydrogels.

As demonstrated by many previous studies, hypoxia and cancer cell metastasis are important factors in many conventional cancer therapies. Therefore, for the first time, we combined two nano particles in one intelligent theragnostic platform based on hydrogel for tumor-targeted drug delivery, and the drugs like DOX or MnO_2_@Ce6 and the genes were released by Dicer enzyme. As illustrated in Figures 2G,H, the loading rate of MnO_2_@Ce6 onto HMC was calculated to be around 2.4 mg/ml.

### *In Vitro* Experiments with HDMC

**FIGURE 3:**
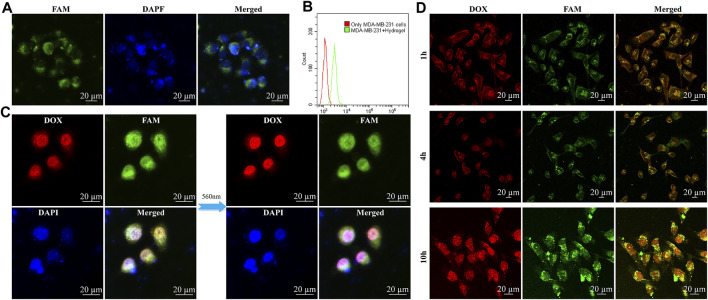
*In vitro* experiments with hydrogel and HD. The confocal microscopic images of targeted MDA-MB-231 cell internalization of FAM-labelled hydrogel. Green, red, and blue signals indicated FAM-labelled hydrogel, DOX, and DAPI dyes, respectively. **(A)** MDA-MB-231 cells incubated with hydrogel for 2 h, the blue one is nucleus, the green one is FAM-labelled hydrogel which signals were around the blue nucleus. **(B)** Based on flow cytometry, detected the intensity of fluorescent signals in cells. **(C)** The difference of the radiation of 560 nm to HD in MDA-MB-231 cells was illustrated (without the radiation of 560 nm and with the radiation of 560 nm for 50 min) and DOX accumulated in nucleus to kill cells. **(D)** These pictures are different time stages of HD which incubate with MDA-MB-231 cells, and we can see the DOX were gathering in nucleus to kill cells: HD incubation with MDA-MB-231 cells for 1, 4, and 10 h.

As revealed in many previous studies, the target of aptamer LXL-1 with the best recognition and selectivity has been determined by Chaoyong James Yang and his team (Li et al., 2014). We designed the LXL apt-chol bond with Rtrs to target MDA-MB-231 cell and compacted the micro-sponge into a small space. In the confocal microscopic images (Figure 3A), we can determine whether the hydrogel is taken up by cells into cytoplasm or cell nucleus. We used DAPI to locate cell nucleus, which had an excitation wavelength of 404 nm and emitted blue signals. The hydrogel modified with FAM on the aptamer and CpG strand emitted green signals around the blue signals, so the hydrogel stayed in cytoplasm and was cleaved by Dicer enzyme to kill the cancer cells. As shown in Figure 3B, based on flow cytometry, we can see that the MDA-MB-231 cells incubated with hydrogel have a lot more fluorescent particles than MDA-MB-231 cells without hydrogel. Besides, the confocal microscopic images of untargeted cell group such as L02 cells (Supplementary Figure S3A) and MCF-7 cells (Supplementary Figure S3B) with the internalized FAM-labelled hydrogel were much darker than the fluorescent intensity of targeted cell (MDA-MB-231). Based on flow cytometry (Supplementary Figures S3C,D), the intensity of fluorescent signals in cells was detected. The confocal microscopic images results demonstrated that the targeted cells had more fluorescent particles than the untargeted cells. In addition, since the hydrogel was produced by the RCT reaction, DOX was introduced into GC bands, and there were many GC bands in hydrogel. Therefore, there were many DOX binding sites. When HD was incubated with MDA-MB-231 cells, DOX accumulated around cell nucleus with time extends or under 560 nm laser radiation for 50 min, as shown in Figure 3C,D. Mean fluorescence intensity (MFI) analysis of DOX in Supplementary Figure S2B also showed that DOX accumulated around cell nucleus.

**FIGURE 4:**
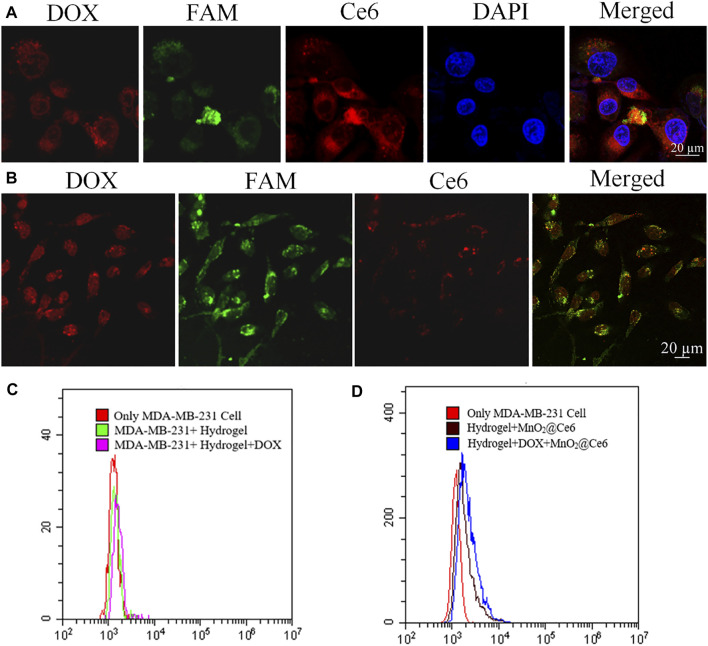
*In vitro* experiments with HDMC. **(A)** Confocal images of MDA-MB-231 cells incubated with HDMC for 2 h with 660-nm laser irradiation (50 mW cm^−2^) for 10 min. **(B)** Confocal images of MDA-MB-231 cells incubated with HDMC for 2 h. **(C, D)** The detected intensity of fluorescent signals of hydrogel, HD, HMC, and HDMC incubated with MDA-MB-231 cells for 2 h and with 660-nm laser irradiation (50 mW cm^−2^) for 10 min in cells with the same concentration based on flow cytometry.

It has been known that MnO_2_@Ce6 is stable under neutral and basic solution but would decompose into Mn^2+^ and O_2_ under acidic environment. Since MnO_2_@Ce6 was unstable in FBS, we combined hydrogel and MnO_2_@Ce6 together to stabilize MnO_2_@Ce6 and enhance the heal effects. As shown in Figure 4, the nucleus was stained with DAPI (excitation wavelength of 404.1 nm, fluorescence detection band of 425–475 nm), CpG strands and aptamer strands were modified FAM and bound on hydrogel. The excitation wavelength was 488 nm, the fluorescence detection band was in the range of 500–550 nm, the DOX excitation wavelength was 560.6 nm, the fluorescence detection band was 570–620 nm, the Ce6 excitation wavelength was 639.8 nm, and the fluorescence detection band was 670–720 nm. As shown in Figure 4A, HDMC was incubated with MDA-MB-231 for 2 h, these cells were treated with 660 nm light (50 mW cm^−2^) for 10 min. Compared with the fluorescence of non-treated Ce6 in Figure 4B, the fluorescence of Ce6 treated with 660 nm light was significantly enhanced. Based on flow cytometry, the intensities of fluorescent signals of hydrogel, HD, HMC, HDMC incubated with MDA-MB-231 cells for 2 h and with 660-nm laser irradiation (50 mW cm^−2^) for 10 min in cells at the same concentration were detected, as shown in Figures 4C,D.

### *Ex Vivo* and *In Vivo* Treatment with HDMC

**FIGURE 5:**
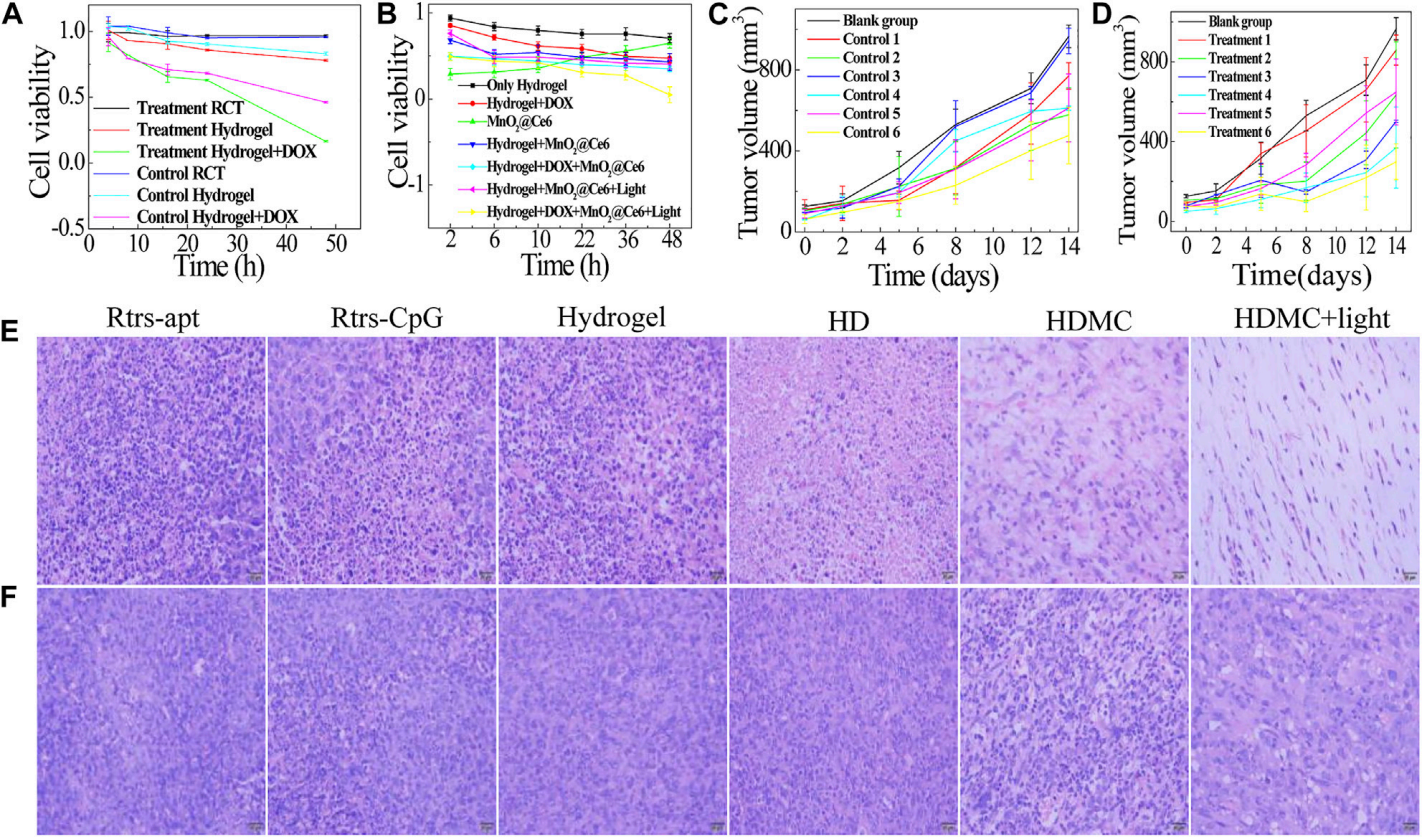
*In vitro* and *in vivo* synergistic therapeutic effects. Relative viabilities of MDA-MB-231 cells after incubation with the different drugs: **(A)** The treatment gene and control gene (random sequence) to carry different drugs; **(B)** The treatment gene carries the different drug under different conditions. **(C, D)** The tumor volume of each group mice (*n* = 3). Group 1 is Rtrs + apt, group 2 is Rtrs + CpG, group 3 is hydrogel, group 4 is HD, group 5 is HDMC, and group 6 is HDMC + Light. H&E-stained tumor slices collected from mice post various treatments indicated: **(E)** treatment group; **(F)** control group (scale bars are 20 μm).

It is known that cancers inside tumors are a large number of dicer enzyme which can gradually cut double-stranded RNA introduced by exogenous sources or transgenic or viral infections in an ATP-dependent manner. And then the drugs delivered to cytoplasm will work. We designed three groups, i.e., blank group which injected TM buffer through the assay, control group which injected scrambled sequence gene, and treatment group which injected treatment gene. As shown in Figure 5A, we designed a control ssDNA against RCT under the same condition and carried different drugs. The toxicity of treatment or control RCT was low because they had large sizes and can hardly enter cells. The hydrogel which bonded to CpG and apt was prone to toxicity to some extent. The most effective treatment was to mix the hydrogel with DOX, and the control group was getting toxic in a sense. Next, we further verified the addition of MC with or without 660-nm laser irradiation (50 mW cm^−2^) for 10 min. As illustrated in Figure 5B, the naked MC had low cell viability due to the high cytotoxicity of PAH. The cell viability of only hydrogel, HD, HMC, HMC + Light, HDMC, HDMC + Light, decreased successively. The trend of therapeutic effect can be confirmed in the following *in vivo* assay. The tumor sizes and mice body weights were measured in following 2 weeks (Figure 5C,D and Supplementary Figure S4). It is worth noting that the growth rate of group 6 with the combination therapy is the slowest, indicating the photodynamic therapy and chemotherapy by delivered hydrogel composed of two treatment gene have significant synergistic effect. In 14 days, the average body weights of Balb/C mice during various treatments stably increased, indicating these treatments did not induce obvious toxic side effects to mice.

### *In Vivo* Combined Gene-Chemo-PDT Treatment with Different Nano Particals

It is known that cancer cells inside tumors can constitutively produce H_2_O_2_, whose level has been reported to be in the range of 10–100 µM in many types of solid tumors. In our analysis, the hematoxylin and eosin (H and E) staining of tumor slices also showed that most of the HDMC + Light tumor cells were severely damaged in the group with 660-nm light (Figure 5E). Figure 5E is a treatment group synthesized with the treatment gene as the carrier and Figure 5F is the scrambled miRNA gene. After 14 days, H&E-stained images of major organs from the combination therapy group suggested that our gene-chemo-PDT induced no obvious toxic side effects to mice. In addition, we all know that the 4T1 cells are easily transferred to lung, and we can see the differences between them in Supplementary Figures S5, S6. As shown in Supplementary Figure S6, there is a certain correlation between the size of tumors and the size of spleen. In fact, the mammary tumour induced with 4T1 cells is known for presenting splenomegaly (DuPre and Hunter, 2007). Besides, the white dot on lungs is the metastatic cancer cell. As shown in Supplementary Figure S6A, due to the synergistic inhibition of metastasis of MnO_2_ and miRNA-182, these three groups of HD, HDMC, HDMC + light have less metastasis, while other groups and Supplementary Figure S6B have more metastasis.

## Conclusion

In this paper, we developed RNA hydrogel as the carrier to deliver targeted drug into MDA-MB-231 cells. RNA hydrogel is the basic component of nanoprobes, and its main effect in our design is to prevent miRNA from being decomposed, and act as a nanocarrier to modify aptamer, DOX, and CpG. Moreover, MnO_2_@Ce6 is loaded on the RNA hydrogel through electrostatic action, which can improve the hypoxic environment of the tumor and enhance PDT and chemotherapy. The developed nano platform can integrate these three gene-chemo-PDT treatment methods into a single carrier, and at the same time, the nucleic acid aptamer can improve the targetability to improve the therapeutic effect. The gene-chemo-PDT nano platform is not only the sum of the curative effect of these three cancer treatments but also complementary to each other. We believe that this highly efficient nano-platform system will have a wide range of potential applications in cancer treatment.

## Data Availability

The original contributions presented in the study are included in the article/Supplementary Material, further inquiries can be directed to the corresponding author.

## References

[B1] Castro N. P. Fedorova-Abrams N. D. Merchant A. S. Rangel M. C. Nagaoka T. Karasawa H. (2015). Cripto-1 as a Novel Therapeutic Target for Triple Negative Breast Cancer. Oncotarget 6 (14), 11910–11929. 10.18632/oncotarget.4182 26059540 PMC4494913

[B2] Chen H. Tian J. He W. Guo Z. (2015). H2O2-Activatable and O2-Evolving Nanoparticles for Highly Efficient and Selective Photodynamic Therapy against Hypoxic Tumor Cells. J. Am. Chem. Soc. 137 (4), 1539–1547. 10.1021/ja511420n 25574812

[B3] Chen Q. Feng L. Liu J. Zhu W. Dong Z. Wu Y. (2016). Intelligent Albumin-MnO2Nanoparticles as pH-/h2o2-Responsive Dissociable Nanocarriers to Modulate Tumor Hypoxia for Effective Combination Therapy. Adv. Mater. 28 (33), 7129–7136. 10.1002/adma.201601902 27283434

[B4] Conde J. Oliva N. Atilano M. Song H. S. Artzi N. (2016). Self-assembled RNA-Triple-helix Hydrogel Scaffold for microRNA Modulation in the Tumour Microenvironment. Nat. Mater 15 (3), 353–363. 10.1038/nmat4497 26641016 PMC6594154

[B5] Cutler J. I. Auyeung E. Mirkin C. A. (2012). Spherical Nucleic Acids. J. Am. Chem. Soc. 134 (3), 1376–1391. 10.1021/ja209351u 22229439

[B6] Ding L. Li J. Wu C. Yan F. Li X. Zhang S. (2020). A Self-Assembled RNA-Triple helix Hydrogel Drug Delivery System Targeting Triple-Negative Breast Cancer. J. Mater. Chem. B 8 (16), 3527–3533. 10.1039/c9tb01610d 31737891

[B7] duPre' S. A. Hunter K. W. Jr. (2007). Murine Mammary Carcinoma 4T1 Induces a Leukemoid Reaction with Splenomegaly: Association with Tumor-Derived Growth Factors. Exp. Mol. Pathol. 82 (1), 12–24. 10.1016/j.yexmp.2006.06.007 16919266

[B8] DuPre´ S. A. Redelman D. Hunter K. W. Jr. (2007). The Mouse Mammary Carcinoma 4T1: Characterization of the Cellular Landscape of Primary Tumours and Metastatic Tumour Foci. Int. J. Exp. Pathol. 88 (5), 351–360. 10.1111/j.1365-2613.2007.00539.x 17877537 PMC2517332

[B9] Gilam A. Conde J. Weissglas-Volkov D. Oliva N. Friedman E. Artzi N. (2016). Local microRNA Delivery Targets Palladin and Prevents Metastatic Breast Cancer. Nat. Commun. 7, 12868. 10.1038/ncomms12868 27641360 PMC5031803

[B10] Gong Y. Ji P. Yang Y.-S. Xie S. Yu T.-J. Xiao Y. (2021). Metabolic-pathway-based Subtyping of Triple-Negative Breast Cancer Reveals Potential Therapeutic Targets. Cel Metab. 33 (1), 51–64. 10.1016/j.cmet.2020.10.012 33181091

[B11] Ismail-Khan R. Bui M. M. (2010). A Review of Triple-Negative Breast Cancer. Cancer Control 17 (3), 173–176. 10.1177/107327481001700305 20664514

[B12] Jang M. Han H. D. Ahn H. J. (2016). A RNA Nanotechnology Platform for a Simultaneous Two-In-One siRNA Delivery and its Application in Synergistic RNAi Therapy. Sci. Rep. 6, 32363. 10.1038/srep32363 27562435 PMC4999871

[B13] Jang M. Kim J. H. Nam H. Y. Kwon I. C. Ahn H. J. (2015). Design of a Platform Technology for Systemic Delivery of siRNA to Tumours Using Rolling circle Transcription. Nat. Commun. 6, 7930. 10.1038/ncomms8930 26246279 PMC4918333

[B14] Ji X. Guo D. Ma J. Yin M. Yu Y. Liu C. (2021). Epigenetic Remodeling Hydrogel Patches for Multidrug‐Resistant Triple‐Negative Breast Cancer. Adv. Mater. 33 (18), 2100949. 10.1002/adma.202100949 33792093

[B15] Krafft M. P. (2020). Alleviating Tumor Hypoxia with Perfluorocarbon-Based Oxygen Carriers. Curr. Opin. Pharmacol. 53, 117–125. 10.1016/j.coph.2020.08.010 32979727

[B16] Lee J. Yesilkanal A. E. Wynne J. P. Frankenberger C. Liu J. Yan J. (2019). Effective Breast Cancer Combination Therapy Targeting BACH1 and Mitochondrial Metabolism. Nature 568 (7751), 254–258. 10.1038/s41586-019-1005-x 30842661 PMC6698916

[B17] Li J. Yuan D. Zheng X. Zhang X. Li X. Zhang S. (2020). A Triple-Combination Nanotechnology Platform Based on Multifunctional RNA Hydrogel for Lung Cancer Therapy. Sci. China Chem. 63 (4), 546–553. 10.1007/s11426-019-9673-4

[B18] Li X. Zhang W. Liu L. Zhu Z. Ouyang G. An Y. (2014). *In Vitro* selection of DNA Aptamers for Metastatic Breast Cancer Cell Recognition and Tissue Imaging. Anal. Chem. 86 (13), 6596–6603. 10.1021/ac501205q 24892693

[B19] Li Y. Xiao Y. Lin H.-P. Reichel D. Bae Y. Lee E. Y. (2019). *In Vivo* β-catenin Attenuation by the Integrin α5-targeting Nano-Delivery Strategy Suppresses Triple Negative Breast Cancer Stemness and Metastasis. Biomaterials 188, 160–172. 10.1016/j.biomaterials.2018.10.019 30352320

[B20] Liu L. Wang Y. Miao L. Liu Q. Musetti S. Li J. (2018). Combination Immunotherapy of MUC1 mRNA Nano-Vaccine and CTLA-4 Blockade Effectively Inhibits Growth of Triple Negative Breast Cancer. Mol. Ther. 26 (1), 45–55. 10.1016/j.ymthe.2017.10.020 29258739 PMC5763160

[B21] Luo Y. (2007). Preparation of MnO2 Nanoparticles by Directly Mixing Potassium Permanganate and Polyelectrolyte Aqueous Solutions. Mater. Lett. 61 (8-9), 1893–1895. 10.1016/j.matlet.2006.07.165

[B22] Mai X. Chang Y. You Y. He L. Chen T. (2021). Designing Intelligent Nano-Bomb with On-Demand Site-specific Drug Burst Release to Synergize with High-Intensity Focused Ultrasound Cancer Ablation. J. Controlled Release 331, 270–281. 10.1016/j.jconrel.2020.09.051 33010331

[B23] Pawar A. Prabhu P. (2019). Nanosoldiers: A Promising Strategy to Combat Triple Negative Breast Cancer. Biomed. Pharmacother. 110, 319–341. 10.1016/j.biopha.2018.11.122 30529766

[B24] Peng T. Deng Z. He J. Li Y. Tan Y. Peng Y. (2020). Functional Nucleic Acids for Cancer Theranostics. Coord. Chem. Rev. 403, 213080. 10.1016/j.ccr.2019.213080

[B25] Prasad P. Gordijo C. R. Abbasi A. Z. Maeda A. Ip A. Rauth A. M. (2014). Multifunctional Albumin-MnO2 Nanoparticles Modulate Solid Tumor Microenvironment by Attenuating Hypoxia, Acidosis, Vascular Endothelial Growth Factor and Enhance Radiation Response. ACS Nano 8 (4), 3202–3212. 10.1021/nn405773r 24702320

[B26] Secret E. Maynadier M. Gallud A. Chaix A. Bouffard E. Gary-Bobo M. (2014). Two-photon Excitation of Porphyrin-Functionalized Porous Silicon Nanoparticles for Photodynamic Therapy. Adv. Mater. 26 (45), 7643–7648. 10.1002/adma.201403415 25323443

[B27] Seeman N. C. (2010). Nanomaterials Based on DNA. Annu. Rev. Biochem. 79, 65–87. 10.1146/annurev-biochem-060308-102244 20222824 PMC3454582

[B28] Shu D. Shu Y. Haque F. Abdelmawla S. Guo P. (2011). Thermodynamically Stable RNA Three-Way junction for Constructing Multifunctional Nanoparticles for Delivery of Therapeutics. Nat. Nanotech 6 (10), 658–667. 10.1038/nnano.2011.105 PMC3189281 21909084

[B29] Song M. Liu T. Shi C. Zhang X. Chen X. (2016). Bioconjugated Manganese Dioxide Nanoparticles Enhance Chemotherapy Response by Priming Tumor-Associated Macrophages toward M1-like Phenotype and Attenuating Tumor Hypoxia. ACS Nano 10 (1), 633–647. 10.1021/acsnano.5b06779 26650065 PMC5242343

[B30] Tao Y. Zhu L. Zhao Y. Yi X. Zhu L. Ge F. (2018). Nano-graphene Oxide-Manganese Dioxide Nanocomposites for Overcoming Tumor Hypoxia and Enhancing Cancer Radioisotope Therapy. Nanoscale 10 (11), 5114–5123. 10.1039/c7nr08747k 29487939

[B31] Wilson W. R. Hay M. P. (2011). Targeting Hypoxia in Cancer Therapy. Nat. Rev. Cancer 11 (6), 393–410. 10.1038/nrc3064 21606941

[B32] Xiao K. Li Y. Luo J. Lee J. S. Xiao W. Gonik A. M. (2011). The Effect of Surface Charge on *In Vivo* Biodistribution of PEG-Oligocholic Acid Based Micellar Nanoparticles. Biomaterials 32 (13), 3435–3446. 10.1016/j.biomaterials.2011.01.021 21295849 PMC3055170

[B33] Yang G. Gong H. Liu T. Sun X. Cheng L. Liu Z. (2015). Two-dimensional Magnetic WS2@Fe3O4 Nanocomposite with Mesoporous Silica Coating for Drug Delivery and Imaging-Guided Therapy of Cancer. Biomaterials 60, 62–71. 10.1016/j.biomaterials.2015.04.053 25985153

[B34] Yin Z. Ji Q. Wu D. Li Z. Fan M. Zhang H. (2021). H2O2-Responsive Gold Nanoclusters @ Mesoporous Silica @ Manganese Dioxide Nanozyme for "Off/On" Modulation and Enhancement of Magnetic Resonance Imaging and Photodynamic Therapy. ACS Appl. Mater. Inter. 13 (13), 14928–14937. 10.1021/acsami.1c00430 33759491

[B35] Yu K.-X. Qiao Z.-J. Song W.-L. Bi S. (2021a). DNA Nanotechnology for Multimodal Synergistic Theranostics. J. Anal. Test. 5 (2), 112–129. 10.1007/s41664-021-00182-z

[B36] Yu K. Hai X. Yue S. Song W. Bi S. (2021b). Glutathione-activated DNA-Au Nanomachine as Targeted Drug Delivery Platform for Imaging-Guided Combinational Cancer Therapy. Chem. Eng. J. 419, 129535. 10.1016/j.cej.2021.129535

[B37] Yue S. Li Y. Qiao Z. Song W. Bi S. (2021). Rolling Circle Replication for Biosensing, Bioimaging, and Biomedicine. Trends Biotechnol. 39 (11), 1160–1172. 10.1016/j.tibtech.2021.02.007 33715868

[B38] Yue S. Song X. Song W. Bi S. (2019). An Enzyme-free Molecular Catalytic Device: Dynamically Self-Assembled DNA Dendrimers for *In Situ* Imaging of microRNAs in Live Cells. Chem. Sci. 10 (6), 1651–1658. 10.1039/c8sc04756a 30842828 PMC6369435

[B39] Zhang W. Guan X. Tang J. (2021). The Long Non‐coding RNA Landscape in Triple‐negative Breast Cancer. Cell Prolif 54 (2), e12966. 10.1111/cpr.12966 33314471 PMC7848969

[B40] Zhu G. Hu R. Zhao Z. Chen Z. Zhang X. Tan W. (2013a). Noncanonical Self-Assembly of Multifunctional DNA Nanoflowers for Biomedical Applications. J. Am. Chem. Soc. 135 (44), 16438–16445. 10.1021/ja406115e 24164620 PMC3855874

[B41] Zhu G. Mei L. Vishwasrao H. D. Jacobson O. Wang Z. Liu Y. (2017). Intertwining DNA-RNA Nanocapsules Loaded with Tumor Neoantigens as Synergistic Nanovaccines for Cancer Immunotherapy. Nat. Commun. 8 (1), 1482. 10.1038/s41467-017-01386-7 29133898 PMC5684198

[B42] Zhu G. Zheng J. Song E. Donovan M. Zhang K. Liu C. (2013b). Self-assembled, Aptamer-Tethered DNA Nanotrains for Targeted Transport of Molecular Drugs in Cancer Theranostics. Proc. Natl. Acad. Sci. 110 (20), 7998–8003. 10.1073/pnas.1220817110 23630258 PMC3657780

[B43] Zhu W. Dong Z. Fu T. Liu J. Chen Q. Li Y. (2016). Modulation of Hypoxia in Solid Tumor Microenvironment with MnO2Nanoparticles to Enhance Photodynamic Therapy. Adv. Funct. Mater. 26 (30), 5490–5498. 10.1002/adfm.201600676

