# Supplementary material for: RNA Hydrogel Combined with MnO2 Nanoparticles as a Nano-Vaccine to Treat Triple Negative Breast Cancer
Source: Front Chem. 2021 Dec 24;9:797094. doi: 10.3389/fchem.2021.797094 (PMC8739783; doi:10.3389/fchem.2021.797094)
Supplement: Supplementary file 1 [file DataSheet1.PDF]

Supplementary Material

RNA Hydrogel combined with MnO_2 Nanoparticles as a Nano-vaccine to Treat Triple Negative Breast Cancer

Weicai Wang[1], Xiaofan Liu[1], Lairong Ding[1], Hyung Jong Jin[2] and Xuemei Li[1*]

[1] Collaborative Innovation Center of Tumor Marker Detection Technology, Equipment and Diagnosis-Therapy Integration in Univer-sities of Shandong, Shandong Province Key Laboratory of Detection Technology for Tumor Makers, School of Chemistry and Chemical Engineering, Linyi University, Linyi, China

[2] Department of Bioscience and Biotechnology, the University of Suwon, Hwaseong City, Gyeonggi-Do, Republic of Korea

***Correspondence:** xuemei_li@yeah.net

Supplementary Table S1. DNA sequence used in this work

No.	Sequence
ssDNA	5'-phosphate- ATAGTGAGTCGTATTA AAAAA **AAA CCG TTA CCA TCT TGA GTG TGA CCA CTC CAT TGT CCT AGG CCA CCA AGA TCT GAA CGG TTGAAAAA**AAG TCA CCT CAC TTC GAA CAG GAA GTA AGG TGG CCT CAG ACGAAAAAATCCCT -3'
ssDNA for scrambled shRNA	5'-phosphate- ATAGTGAGTCGTATTA **AAAAA GGA CAA CTGCCA TCG CCG TCA CTG ATA TTT CAT GAT TCT ACT AGG GAT TCC GCC ACA GGA CATAAAAA**GCT GAG GAA AGT CCA GTG AAC GAA CAT ACC CTA GCG TGA CCTAAAAAATCCCT -3'
CpG	5'-**FAM** -**AAA** ATCCC TATAG TGAGT CGTAT TA **AAA**TCC ATG ACG TTC CTG ACG TT---**Chol** -3'
T7 promotor	5'-TAA TAC GAC TCA CTA TAG GGA T -3'
F-C-LXL apt	5'-**FAM**- **CAC TCC ATT GTC CTA GGC**GAA TTC AGT CGG ACA GCG AAG TAG TTT TCC TTC TAA CCT AAG AAC CCG CGG CAG TTT AAT GTA GAT GGA CGA A - **Chol** -3'

The ssDNA sequence marked in red will bind to T7 promotor sequence. Bold blue sequence in ssDNA is shRNA-182 sequence, which will bind to the blue one in Fam-Circle of shmiR-182 steam loop-LXL apt to modify an aptamer of MDA-MB-231 cells. The random sequence marked in green is the site for Dicer. The sequence marked in brown is shRNA-205 sequence. The part of CpG sequence which are circled in gay will bind to the part of ssDNA sequence circled in gay.

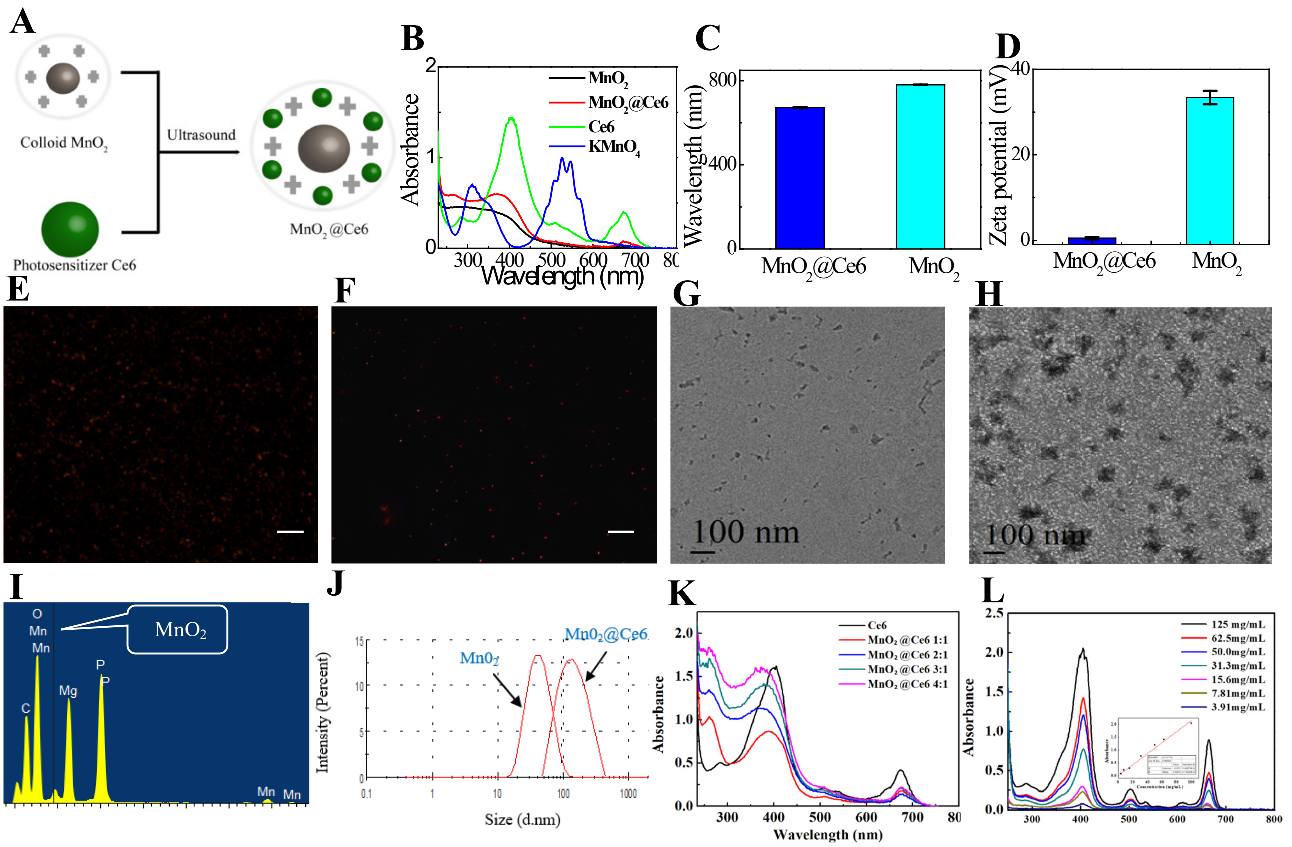

Supplementary Figure S1. Synthesis and characterize of MnO_2@ Ce6 and MnO_2. (A) Illustration of the synthesis of MnO_2@ Ce6 nanoparticles. (B) UV–vis spectra of Ce6, $KMnO_4$, MnO_2, and MnO_2@Ce6 nanoparticles. (C) Scattering spectrometer of MnO_2@ Ce6 and MnO_2. Their maximum scattering peaks of MnO_2@ Ce6and MnO_2 were different. (D) Zeta potential of MnO_2@ Ce6 and MnO_2. (E, F) Dark-field microscopy of MnO_2@ Ce6 and MnO_2 (scale bars are 20 μm). (G, H) TEM of MnO_2@ Ce6 and MnO_2. (I) EDS elemental analysis of MnO_2@ Ce6. (J) The size of MnO_2@ Ce6 and MnO_2. (K) The supernatant of 20 μL, 40 μL, 60 μL, and 80 μL colloidal MnO_2 was added into 20 μL of activated Ce6 (10 mg/mL) with ultrasonication for 4 h and the UV-vis spectrum was obtained. When MnO_2: Ce6 was 4:1, the MnO_2@ Ce6 was stable in DI water. (L) The linear equation of different concentrations of Ce6 is y=0.025x-0.001 and the loading rate of Ce6 in MnO_2 is around 66.4 mg/mL.

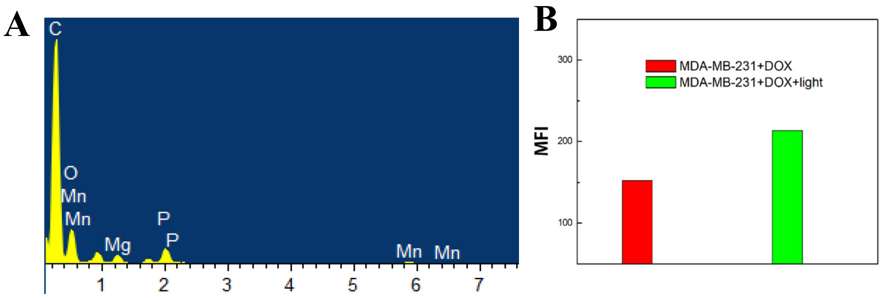

Supplementary Figure S2. (A)EDS elemental analysis of HMC. (B)Mean fluorescence intensity (MFI) analysis of DOX in Figure 3c.

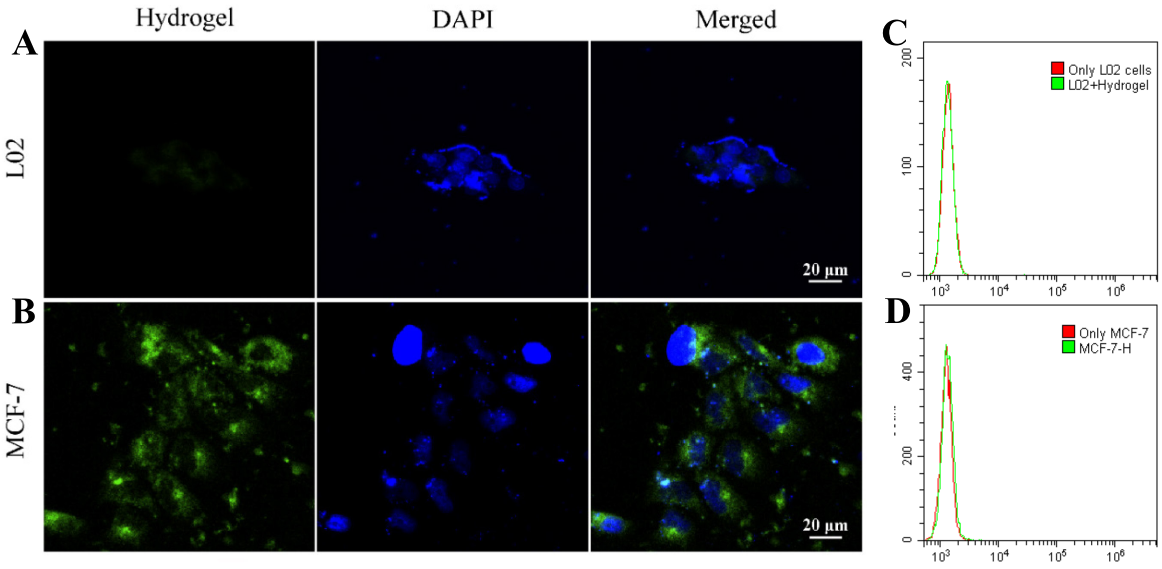

Supplementary Figure S3. The confocal microscopic images of untargeted cell group such as (A) L02 cells and (B) MCF-7 cells internalizing FAM-labeled Hydrogel (scale bars are 20 μm). Green and blue signals indicate FAM-labeled Hydrogel and DAPI dyes, respectively. Based on Flow cytometry (C, D), the intensity of fluorescent signals in cells was detected.

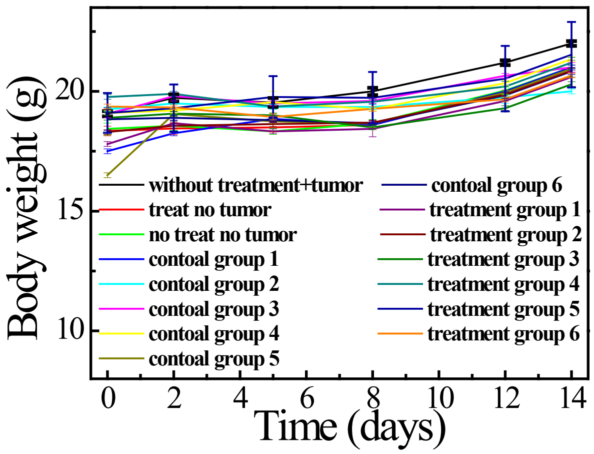

Supplementary Figure S4. Average body weights of Balb/C mice during various treatments. Error bars were calculated from the standard errors of the mean (n =3).

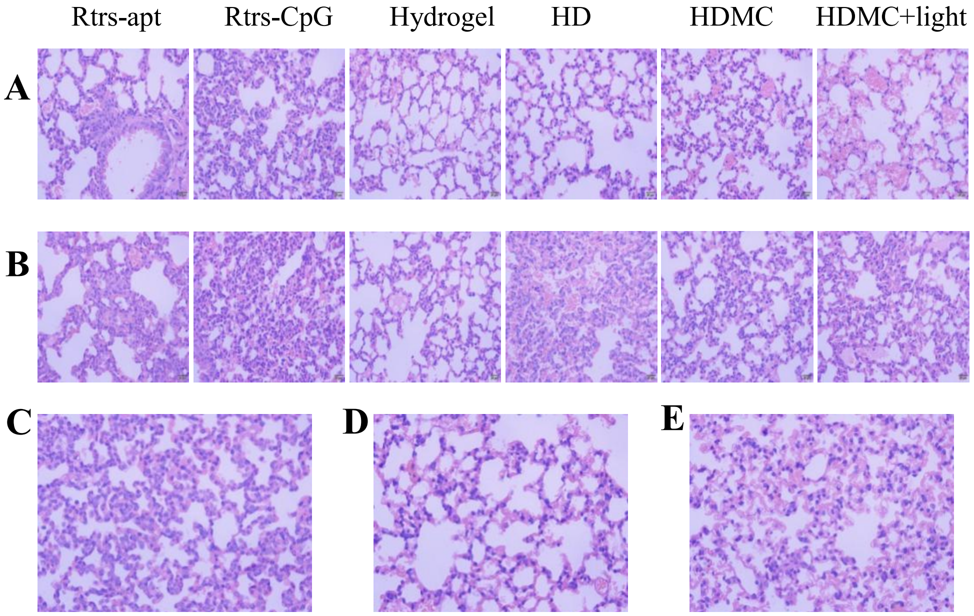

Supplementary Figure S5. The lungs of these Balb/C mice after various treatments. (A) The treatment group; (B) The control group; (C) With tumor but no treatment; (D) No tumor but with treatment; (E) Normal mice. Scale bars are 20 μm.

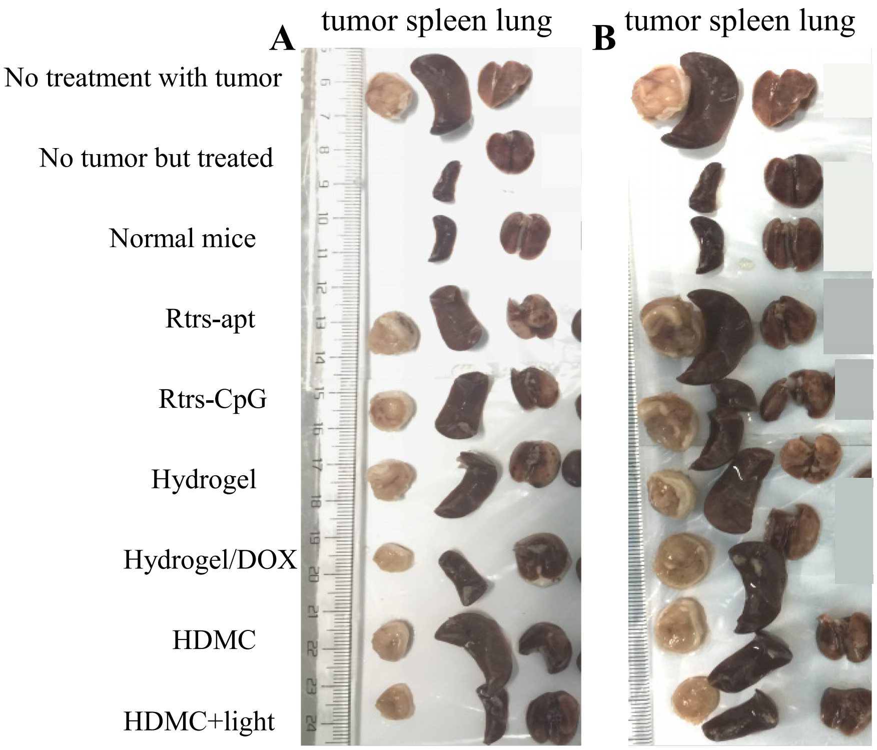

Supplementary Figure S6. Ex vivo images of tumors, spleens, and lungs depicted in treated mice. (A) Treatment group; (B) Control group.
